# Study on the Synchronous Removal of Nitrogen and Phosphorus by Autotrophic/Heterotrophic Denitrification in the Presence of Pyrite

**DOI:** 10.3390/molecules30112412

**Published:** 2025-05-30

**Authors:** Minyi Zhu, Minhui Ma, Shuo Chen, Rongfang Yuan, Shaona Wang

**Affiliations:** Department of Environmental Science and Engineering, School of Energy and Environmental Engineering, University of Science and Technology Beijing, Beijing 100083, China; zhuminyi177@163.com (M.Z.); minhuima1234@163.com (M.M.); 19003283038@163.com (S.C.)

**Keywords:** autotrophic denitrification, nitrogen and phosphorus removal, pyrite, biofilter, microbial characteristics

## Abstract

Pollution caused by N and P is a significant contributor to water eutrophication. While traditional biological treatment processes can remove some N and P elements from water, the effluent quality often fails to meet the stringent requirements of sensitive areas. The autotrophic denitrification’s simultaneous nitrogen and phosphorus removal pro-cess, known for its low operating cost and minimal sludge production, has garnered considerable attention from researchers. In this study, natural pyrite was used for the removal of nitrogen and phosphorus in a denitrification system, and the underlying mechanisms were elucidated. The results indicate that the N and P removal efficiency was influenced by empty bed contact time (EBCT) and the pH value. The highest NO_3_^−^-N removal rate of 90.24% was achieved at an EBCT of 8 h, while the PO_4_^3−^-P removal rate reached 81.58% at an EBCT of 12 h. The addition of a carbon source enhanced the synergistic autotrophic/heterotrophic denitrification, significantly improving phosphorus removal with an increasing C/N ratio. Microbial characteristics analysis revealed that, at the phylum level, *Chlorobiota*, *Bacteroidota*, and *Chloroflexota* played a crucial role in heterotrophic autotrophic denitrification. At the genus level, *Thauera*, *Aridibacter*, and *Gemmatimonas* were key players in heterotrophic denitrification, while *Thiobacillus*, *Rhodoplanes*, and *Geobacter* were associated with autotrophic denitrification.

## 1. Introduction

The discharge of sewage containing N and P is a primary cause of water eutrophication. Traditional biological treatment processes can remove some N elements from water, but the effluent quality often fails to meet the required standards. Therefore, it is necessary to implement advanced treatment processes to achieve nitrogen and phosphorus removal.

Currently, biological denitrification processes are commonly used to remove NO_3_^−^-N from water. Based on the carbon sources required by microorganisms, biological denitrification technology can be divided into heterotrophic and autotrophic denitrification technologies. Heterotrophic denitrification uses organic matter in sewage as an electron donor to reduce nitrate to nitrogen under anaerobic or anoxic conditions. However, the low carbon-to-nitrogen (C/N) ratio in domestic sewage effluent often necessitates the addition of extra carbon sources, which can lead to secondary pollution, high treatment costs, and the generation of by-products [1,2].

Autotrophic denitrification, on the other hand, uses reducing inorganic H_2_, elemental S (S^0^) [3] and reducing Fe (Fe^0^/Fe^2+^) [4] as electron donors for autotrophic bacteria, with HCO_3_^−^ or CO_3_^2−^ serving as inorganic carbon sources to reduce nitrate to nitrogen. This process has the advantage of producing less residual sludge and secondary pollution due to fewer microbial metabolites, making it a promising technology for wastewater treatment.

Sulfur autotrophic denitrification utilizes elemental sulfur, sulfide, and other substances as electron donors for denitrification, reducing NO_3_^−^-N to N_2_ [5]. Soares [6] used granular S^0^ as the up-flow column filling and NaHCO_3_ as the carbon source to achieve NO_3_^−^-N removal in water at a denitrification rate of 200 gN/(m^3^·d). Dissolved H_2_S and solid sulfides can also be used as electron donors for autotrophic denitrification [7].

Fe autotrophic denitrification involves reducing NO_3_^−^-N or NO_2_^−^-N to gaseous nitrides under anaerobic conditions using Fe^0^ or Fe^2+^ as electron donors. The application of Fe compounds in sludge can form FePO_4_ precipitates with PO_4_^3−^ pollutants, enabling the recovery and utilization of iron-containing sludge and simultaneous nitrogen and phosphorus removal [8]. Zhou et al. [9] used Fe(II) as electron donor to achieve Fe-type denitrification in an up-flow biological filter, and the N removal performance was close to 90%.

In recent years, biological filters filled with natural pyrite and pyrrhotite have been found to not only improve N removal rates but also exhibit good P removal effects [10]; pyrite-based autotrophic denitrification can remove P through chemical precipitation [11]. Denitrifying phosphorus removal (DPR) technology, which leverages the “dual utilization of one carbon source” characteristic of denitrifying phosphorus-accumulating organisms (DPAOs), is considered a promising wastewater treatment technology. Compared to traditional wastewater treatment processes, DPR avoids carbon source competition between denitrifying bacteria and phosphorus-accumulating bacteria (PAOs) and the contradiction of solid retention time (SRT), saving 50% of the carbon source, 30% of aeration cost, and 50% of sludge production [12,13].

In this study, an autotrophic denitrifying N and P removal system was constructed, using pyrite as a filler for autotrophic denitrification, and the effects of empty-bed contact time (EBCT), pH value, and C/N ratio on pollutant removal were investigated. Combined with high-throughput sequencing technology, the microbial community under different working conditions was studied, and the main functional flora in the transformation process of Fe, N, and P were analyzed, revealing the mechanism of simultaneous nitrogen and phosphorus removal in the autotrophic denitrification filter. The study laid a theoretical foundation for the application of autotrophic denitrification technology for nitrogen and phosphorus removal in water treatment.

## 2. Materials and Methods

### 2.1. Experimental Reagents and Devices

All chemicals used in this study were of analytical grade purity. Anhydrous sodium acetate (CH_3_COONa), potassium chloride (KCl), ferric sulfate (FeSO_4_), Sodium hydroxide (NaOH), hydrochloric acid (HCl), sodium bicarbonate (NaHCO_3_), magnesium sulfate (MgSO_4_), and calcium chloride (CaCl_2_) were purchased from Sinopharm Chemical Reagent Co., LTD. (Shanghai, China). Ammonium chloride (NH_4_Cl), manganese chloride tetrahydrate (MnCl_2_·4H_2_O), and potassium nitrate (KNO_3_) were bought from Shanghai Aladdin Biochemical Technology Co., LTD. (Shanghai, China). Potassium dihydrogen phosphate (KH_2_PO_4_), and ethylenediamine tetraacetic acid (EDTA) were obtained from Beijing Chemical Works (Beijing, China). Sodium chloride (NaCl) was sourced from Tianjin Guangfu Technology Development Co., LTD. (Tianjin, China).

The pyrite (FeS_2_) used in this study was sourced from Guilin, Guangxi, with a purity of over 90% and minor impurities such as quartz and calcium carbonate. The filler was screened to obtain particles of approximately 0.25–0.35 mm in size. The ore particles were soaked in a 1% (*v/v*) dilute HCl solution for 2 h to remove surface oxides, washed with deionized water until the pH of the leaching solution was neutral, dried in a drying dish, sealed, stored, and sterilized under ultraviolet irradiation.

CH_3_COONa, KNO_3_, NH_4_Cl and KH_2_PO_4_ were used prepared the test water by adjusting the COD, NO_3_^−^-N, PO_4_^3−^-P, TN and NH_4_^+^-N to be 15.23–61.84, 12.36–26.73, 0.74–1.56, 13.65–27.51 and 0.81–2.34 mg/L, respectively. In the test water, MgSO_4_·7H_2_O (0.2 g/L), KCl (0.434 g/L), CaCl_2_ (1.602 g/L) and NaHCO_3_ (21.02 g/L) were also added, with an influent pH of 5–9. The inoculated sludge was taken from the anoxic zone of a reclaimed water plant, screened through a 10-mesh screen, and left for 3 days. The sludge was pretreated to remove interfering ions before being inoculated into the experimental conical bottle. A specific quantity of activated sludge was retrieved from the sludge storage container and transferred into a beaker. After allowing it to settle for 30 min, the supernatant was discarded. Subsequently, a pre-prepared 0.7% NaCl solution was added to the sludge, followed by another 30 min settling period. This cleaning process, including settling and supernatant removal, was repeated three times. For the final step, the sludge was left to sit overnight to drain the supernatant once more, preparing it for inoculation.

**Figure 1:**
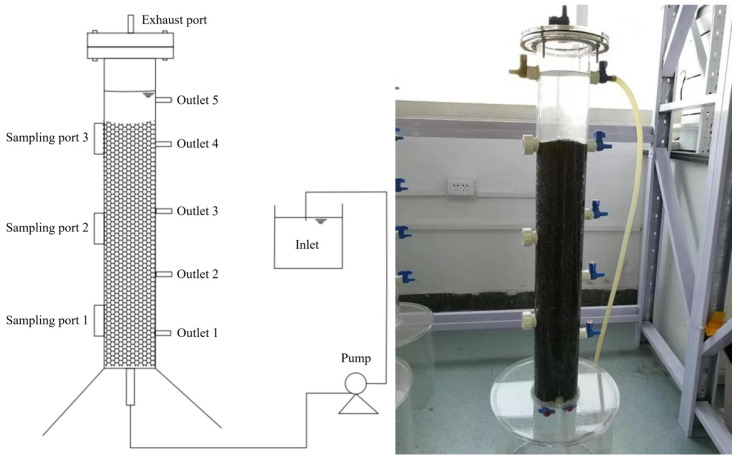
The schematic figure and photo of the bioreactor.

An up-flow biofilter reactor was used (Figure 1). The material of the reactor is cylindrical plexiglass, the total height is 100 cm, the effective height is 80 cm, the inner diameter is 8 cm, the effective volume is about 4 L, the pyrite filling area is 65 cm, and the filling volume is about 3.2 L. The water is continuously fed from the bottom of the reactor by the distribution bucket through the peristaltic pump, and the water outlet at the top is discharged. The taking port is 20 cm apart, and the upper end is provided with an exhaust port.

### 2.2. Reactor Start-Up and Parameter Optimization

Pyrite was loaded into the reactor, and the membrane was hung to run for 30 days. The success of the membrane hanging was evaluated by measuring the inlet and outlet water TN, COD, and PO_4_^3−^-P. The effects of EBCT (12, 8, 4 and 2 h), pH (5, 6, 7, 8 and 9), and C/N (2, 3 and 4) on N and P removal were investigated.

The concentrations of various N and P species, including NO_2_^−^-N, NH_4_^+^-N, NO_3_^−^-N and PO_4_^3−^-P in water, were determined using specific methodologies. For TN, the alkaline potassium persulfate digestion method followed by ultraviolet spectrophotometry was employed, adhering to the Chinese standard HJ 636-2012 [14]. The potassium dichromate method was utilized for COD analysis, conforming to the Chinese standard HJ 828-2017 [15]. The concentration of PO_4_^3−^-P was determined using continuous flow-ammonium molybdate spectrophotometry, in accordance with the Chinese standard HJ 670-2013 [16]. For NO_3_^−^-N, phenoldisulfonic acid spectrophotometry was applied, following the Chinese standard GB 7480-87 [17]. Additionally, spectrophotometric methods were used for NH_4_^+^-N (HJ 535-2009 in China [18]) and NO_2_^−^-N (GB 7493-87 in China [19]).

### 2.3. High Throughput Sequencing and Microbial Diversity Analysis

The iron-containing mineral materials were taken from different sampling layers. The mineral materials were then placed in sterile phosphate buffer solution, vigorously shaken for 15 min to separate the biofilm, and stored in a cryogenic freezer at −86 °C for preservation. Subsequently, DNA samples were extracted from the biofilm, with each sample being replicated three times. Subsequently, amplification was conducted using the V3–V4 region of bacterial 16S rRNA and the primers 341F and 806R. Sequencing was performed with the NovaSeq kit from Shanghai Shenggong Biotechnology Co., LTD. (Shanghai, China), on the Illumina HiSeq 2500 platform. Based on the OTU clustering analysis results, a variety of diversity index analyses can be carried out. Additionally, statistical analysis of the community structure at each taxonomic level can be conducted using the classification information.

## 3. Results and Discussion

### 3.1. Impact of EBCT on N Removal

**Figure 2:**
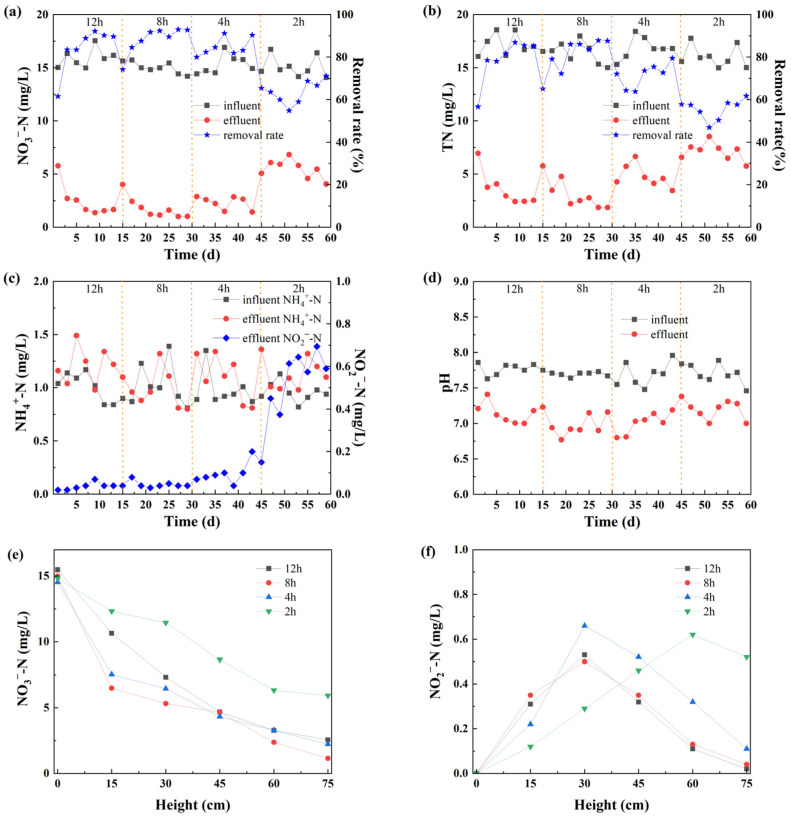
Effect of EBCT on the removal of (**a**) NO_3_^−^-N, (**b**) TN, (**c**) NH_4_^+^-N, NO_2_^−^-N, and (**d**) pH, and the effect of the height of medium level on (**e**) NO_3_^−^-N and (**f**) NO_2_^−^-N removal.

The nitrogen removal efficiency of the denitrification system is significantly influenced by the EBCT, pH values and the height of the filling material. Figure 2 illustrates the N removal effects under different EBCT conditions.

#### 3.1.1. N Removal Rates

Figure 2a shows the impact of EBCT on the removal of NO_3_^−^-N. When the influent NO_3_^−^-N concentration was 15 mg/L and natural pyrite was used as the electron donor filler, significant NO_3_^−^-N removal was achieved. In Stage I, with an EBCT of 12 h, the NO_3_^−^-N removal rate fluctuated, initially increasing from 59.23% to 84.21% and then decreasing to 71.45%. In Stage II, with an EBCT of 8 h, the removal rate increased from 71.45% to 90.24% and then decreased to 79.87%, indicating that even after a short adaptation period, the influent NO_3_^−^-N could still be completely removed. Several factors affect mass transfer and biodegradation in pyrite-based autotrophic denitrification biofilters, including biofilm thickness and fluid velocity [20]. Decreasing the fluid velocity (increasing residence time) can improve packed bed bioreactor performance by allowing more time for reactions to occur and reducing washout of fixed film and suspended biomass [21]. Conversely, it has also been shown that increasing fluid velocity (decreasing residence time) improves mass transfer of substrates to the biofilm, which may also enhance reaction efficiency [21]. In this study, NO_3_^−^-N removal was better at the EBCT of 8 h compared to 12 h, indicating that the system had stabilized, at which time the electron donor release rate, microbial reaction rate and hydraulic residence time reached a better match. Shortening the EBCT from 12 h to 8 h had no significant impact on the autotrophic denitrification system. In Stage III, with an EBCT of 4 h, the removal rate slightly declined to 84.83%, suggesting that the system retained effective denitrification performance and exhibited a certain resilience to hydraulic loading shocks. Nevertheless, the gradual approach to the system’s treatment capacity threshold became evident. In Stage IV, with an EBCT of 2 h, the removal rate significantly decreased to 55.12% and then increased to 63.45%, indicating a substantial impact on the system. Operating at a lower EBCT had a negative impact on the performance of the bioreactor. This is due to the higher NO_3_^−^-N loading rate caused by the shorter EBCT and the limited mass transfer rate of NO_3_^−^-N to the denitrifying reactor [7]. In addition, the higher shear stresses, caused by increasing the flow rate applied to reduce the EBCT, could detach some biofilm and reduce the amount of active biomass in the system [22]. Blindly lengthening or shortening the EBCT is not beneficial for NO_3_^−^-N removal [23], and an 8 h EBCT is optimal in this study.

Figure 2b shows the TN removal effect under different EBCT conditions. With an influent TN concentration of around 17 mg/L, good TN removal was observed. In Stage I, with an EBCT of 12 h, the TN removal rate fluctuated, increasing from 58.73% to 83.45% and then decreasing to 62.34%. In Stage II, with an EBCT of 8 h, the removal rate increased from 62.34% to 87.4% and then decreased to 72.78%, indicating that after a short adaptation period, the influent TN could still be completely removed. Despite the reduced hydraulic retention time, the system maintained high TN removal efficiency, indicating that the microbial community had adapted to the increased hydraulic loading and sustained a high nitrogen transformation rate under these conditions. Shortening the EBCT from 12 h to 8 h had no significant impact on the system. Compared to Stage I, Stage II exhibited higher and more stable removal performance, suggesting that an EBCT of 8 h provided a better balance between hydraulic conditions and biochemical reactions. The moderate increase in flow rate likely enhanced substrate diffusion to the biofilm without causing excessive biomass washout, thereby promoting stable pyrite-based autotrophic denitrification [15,24]. In Stage III, with an EBCT of 4 h, the removal rate dropped to 62.45%, showing a minor impact. Despite the reduction in efficiency, the system maintained a basic functional TN removal capability. At this shorter contact time, denitrification remained functional but less complete, likely due to the partial accumulation of intermediates (NH_4_^+^-N and NO_2_^−^-N) and the system approaching its kinetic limits [7]. In Stage IV, with an EBCT of 2 h, the removal rate significantly decreased to 45.12% and then increased to 61.52%, indicating a substantial impact on the system. The significant decline in this stage indicates that the system is highly sensitive to extremely short EBCT conditions. The reduced TN removal efficiency is likely associated with the decrease in NO_3−_-N removal and the accumulation of intermediate products. The EBCT of 8 h achieved a high TN removal rate (with a peak value of 87.40%) while maintaining good system stability and can therefore be considered the optimal hydraulic operating parameter for TN removal in this study.

As shown in Figure 2c, the difference between the effluent NH_4_^+^-N concentration and the influent was small. The main reason is that the anoxic environment of the reactor could not provide enough oxygen for nitrifying bacteria, and the abundance of nitrifying bacteria was low, preventing the conversion of NH_4_^+^-N into other forms. Additionally, when the denitrification of NO_2_^−^-N and NO_3_^−^-N was incomplete, they were converted into NH_4_^+^-N, resulting in its accumulation [25]. This explains why the effluent NH_4_^+^-N concentration might be a little higher than the influent. The EBCT may affect the concentration of NO_2_^−^-N in the effluent. As the EBCT was 2 h, partial accumulation of effluent NO_2_^−^-N occurred, with the concentration ranging from 0.35 mg/L to 0.7 mg/L. Under a longer residence time, the contact time between the sewage and the microorganisms in the reactor was longer, allowing anaerobic denitrifying bacteria to fully convert NO_3_^−^-N into N_2_ and NH_4_^+^-N. In addition, due to the long-term residence of the sewage, the trace dissolved oxygen in the influent re-oxidized NO_2_^−^-N into NO_3_^−^-N, leading to the decrease in the NO_2_^−^-N accumulation. Similar results were obtained in the research of Matsubayashi et al. [26]; when the EBCT was extended from 2 h to 4 h, the NO_2_^−^-N content could be reduced. Therefore, changing the EBCT would have an impact on some parts of nitrification.

In conclusion, the removal effects of NO_3_^−^-N and TN exhibit similar trends under different EBCT. When the EBCT ranged from 8 to 12 h, a relatively high removal rate can be achieved under suitable concentrations of NO_3_^−^-N and TN. However, the impact of EBCT on the removal rate varies. When the EBCT was 4 h, its influence on the removal rate was relatively minor; in contrast, when the EBCT was 2 h, its impact on the removal rate was more pronounced. During the reaction process, there is a certain accumulation of NH_4_^+^-N and NO_2_^−^-N. The NO_3_^−^-N removal rate reached its peak when the EBCT was 8 h. Under this hydraulic retention time, the conversion of N forms within the reactor was optimized for the removal of NO_3_^−^-N.

#### 3.1.2. Changes of pH Values

The value of pH is a crucial factor influencing the metabolic processes of microorganisms. Figure 2d shows the changes in the influent and effluent pH under different EBCT conditions. The influent pH was between 7.5 and 8.0, while the effluent remained relatively stable at 6.8 to 7.5. Research has indicated that a pH range of 6.5–8.0 was more suitable for the growth of *Thiobacillus denitrificans*; when the influent pH is too high or too low, the activity of *T. denitrificans* is inhibited, affecting the removal of NO_3_^−^-N and leading to the accumulation of NO_2_^−^-N [27]. When no additional alkalinity was added to the influent, the effluent pH at each stage of the reactor did not continuously decline and even remained above 6.8. This confirms that the autotrophic denitrification system does not require an external pH buffer, while siderite can supplement the alkalinity [28]. The sulfur autotrophic denitrification process might lead to an increase in the pH of the effluent. In this study, pyrite was filled into the autotrophic denitrifying biofilter, which contains reduced sulfur and can be used as a sulfur source to promote sulfur autotrophic denitrification [29]. Pyrite could buffer the H^+^ generated during the S-autotrophic denitrification process to some degree, maintaining the stability of the system’s pH. The H^+^ generated during the denitrification process promotes the dissolution of siderite, and the generated HCO_3_^−^ can form a buffer system to maintain an almost constant pH. Meanwhile, during the filtration process, due to the presence of iron ions in the ore, the pH in the reactor is relatively low.

#### 3.1.3. Influence of the Height of Medium Level

Figure 2e shows that the effluent NO_3_^−^-N concentration decreased continuously with increasing height. When the EBCT was 2 h, the concentration decline rate was slower, and the removal rate reached its highest value in the range of 30–60 cm. Therefore, the anaerobic environment at this time is conducive to the denitrification process. However, when the EBCT was 8 h, the effluent NO_3_^−^-N concentration was the lowest, regardless of the reactor’s height, indicating that an EBCT of 8 h was a more suitable condition. For NO_2_^−^-N, as shown in Figure 2f, at different EBCT conditions, the effluent NO_2_^−^-N concentration first increased and then decreased, reaching the highest at 30 cm, with concentrations of 0.52 mg/L (EBCT 12 h), 0.48 mg/L (EBCT 8 h), 0.68 mg/L (EBCT 4 h), and 0.6 mg/L (EBCT 2 h), respectively. Combining this with the effluent concentration of NO_3_^−^-N, it can be known that the removal rate of NO_3_^−^-N was the highest at 30 cm, and part of the NO_3_^−^-N was converted into NO_2_^−^-N, resulting in the accumulation of NO_2_^−^-N. When the NO_2_^−^-N accumulates to a certain extent, it begins to inhibit the reduction of NO_3_^−^-N in the denitrification process [30]. Therefore, at 60–75 cm of the reactor, the removal effect of NO_3_^−^-N gradually decreased, and the effluent concentration of NO_2_^−^-N decreased simultaneously. In conclusion, when the EBCT was 8 h, the reactor had the best along-the-way removal effect of NO_3_^−^-N, and the removal rate reached its peak at the height of 30 cm. At this time, the accumulation of NO_2_^−^-N concentration also reached its peak. Subsequently, its accumulation effect inhibited the denitrification process, causing the removal rate of NO_3_^−^-N to decline.

### 3.2. Impact of pH Value on N and P Removal

**Figure 3:**
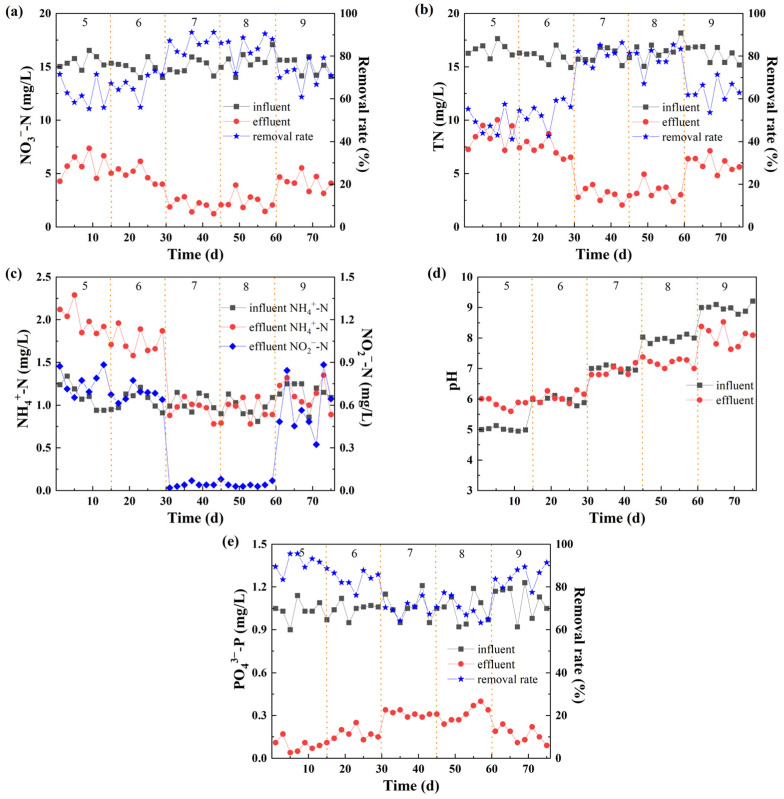
Effect of pH value on the removal of (**a**) NO_3_^−^-N, (**b**) TN, (**c**) NH_4_^+^-N, NO_2_^−^-N, (**d**) pH value, and (**e**) PO_4_^3−^-P.

The pH value plays a significant role in the denitrification process, particularly affecting the activity of related bacteria. Research has indicated that the optimal pH range for denitrification is between 7 and 8 [31]. When the pH exceeds 8.6, it can lead to a decrease in the removal rate of NO_3_^−^-N and an accumulation of NO_2_^−^-N. The effects of pH on the autotrophic denitrification in the reactor are illustrated in Figure 3.

#### 3.2.1. N Removal

As shown in Figure 3a,b, under pH values of 5, 6, 7, 8, and 9, the removal rates of TN and NO_3_^−^-N exhibit similar trends. When the pH was 5, 6, and 9, the average removal rate of NO_3_^−^-N ranged from 55% to 79%, indicating relatively poor overall removal efficiency. However, at pH values of 7 and 8, the maximum removal rate of NO_3_^−^-N reached 91.23%, which is conducive to the denitrification process. The TN removal rate was lower at pH values of 5 and 6. This may be due to the partial conversion of NO_3_^−^-N to NO_2_^−^-N, resulting in its accumulation and thus a lower TN removal rate. When the pH was increased to 7 and 8, the TN removal rate of the system rapidly increased, with an average removal rate reaching 81%. This indicates that denitrifying bacteria began to function more effectively, using NO_2_^−^-N and NO_3_^−^-N as electron acceptors to generate N_2_ [32]. Research has shown that the sulfur autotrophic denitrification process produces acid, and the acid production effect increases with the removal of NO_3_^−^-N [33]. Therefore, an appropriate alkaline condition is beneficial for the removal of NO_3_^−^-N.

As shown in Figure 3c, the average influent NH_4_^+^-N concentration was 1.08 mg/L. The effluent NH_4_^+^-N concentration was relatively high at pH values of 5 and 6, and it gradually decreased as the reactor operated. On the 30th day, after adjusting the pH to 7 or 8, the effluent NH_4_^+^-N concentration dropped significantly. The effluent NO_2_^−^-N concentration continuously accumulated and stabilized at 1.46 mg/L within 0–30 days. When the pH increased to 7 or 8, the pH of the reactor reached the optimal condition for denitrifying bacteria, the denitrification effect improved, and the concentrations of NO_2_^−^-N and NH_4_^+^-N decreased. Specifically, neither NO_2_^−^-N nor NH_4_^+^-N accumulated at this stage, illustrating that the activity of autotrophic denitrifying bacteria was high, and the denitrification reaction proceeded thoroughly. When the pH was further increased to 9, the effluent NO_2_^−^-N concentration increased rapidly, and the rate of NO_2_^−^-N reduction to nitrogen also increased.

#### 3.2.2. P Removal

Figure 3d shows a significant difference in pH values between the influent and effluent. When the influent pH was 5 and 6, the effluent pH increased. This may be due to the accumulation of NO_2_^−^-N at this time, and the heterotrophic denitrification in the system being the main denitrification process, generating more alkalinity and causing the pH to increase [34]. When the pH was further increased to 7 and 8, the effluent pH was lower than the influent, and the pH at this stage was maintained at around 7.1. Combining this with the fact that the NO_2_^−^-N concentration decreased at this time, it indicates that autotrophic denitrification mainly occurred at this stage. The production of acid during the conversion of NO_2_^−^-N to nitrogen led to a decrease in the effluent pH. At the same time, due to the inability to maintain an absolutely anaerobic environment during the filling of materials and the influent process, the metal ions and organic matter in the ore were oxidized, releasing hydrogen ions into the water, resulting in a decrease in the pH value.

Figure 3e reveals that the reactor had a relatively high removal rate of PO_4_^3−^-P at each pH stage, with an average removal rate of 81.2%. The removal effect of PO_4_^3−^-P reached its highest value at 95.61% when the pH was 5, which is mainly related to the precipitation of iron phosphate. When the pH was 9, the average removal effect was 85.26%. Under this pH condition, hydroxide ions combine with iron ions to form P adsorption [35]. During the denitrification process, the possibility of using PO_4_^3−^-P as an electron donor is low. No phosphorus-removing bacteria were found based on microbial community analysis. Therefore, the most likely explanation is that under certain pH conditions, the iron ions in the iron ore combine with PO_4_^3−^-P to form a precipitate, thereby achieving the removal of PO_4_^3−^-P. At the same time, the microbial analysis results show that there are Bacillus in the system. Bacillus has the function of promoting nitrification [36], and part of NO_2_^−^-N can be converted into NO_3_^−^-N, providing an electron donor for dephosphorization by denitrifying microorganisms. In summary, the iron ions in the filler undergo chemical reactions with PO_4_^3−^-P under different pH conditions to produce iron phosphate precipitate, which is used for the removal of PO_4_^3−^-P in the system.

### 3.3. Impact of C/N on N and P Removal

**Figure 4:**
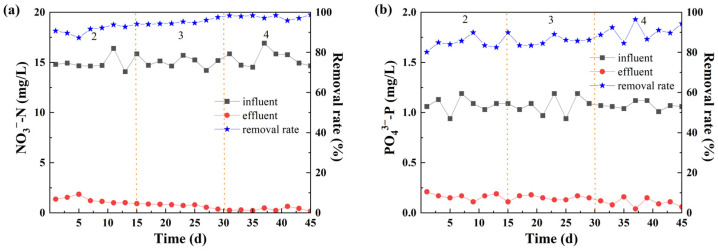
Effect of C/N on (**a**) NO_3_^−^-N and (**b**) PO_4_^3−^-P removal.

The C/N ratio is one of the influencing factors of the autotrophic denitrification system. The addition of a carbon source can effectively promote autotrophic/heterotrophic synergistic denitrification to a certain extent. The nitrogen and phosphorus removal effects of the system under different C/N ratios are shown in Figure 4.

#### 3.3.1. NO_3_^−^-N Removal

An autotrophic/heterotrophic synergistic denitrification system was constructed by adding sodium acetate. When the C/N ratios were 2, 3, and 4, the removal rates of NO_3_^−^-N were 97.23%, 97.92%, and 99.58%, respectively. The addition of a carbon source can promote autotrophic/heterotrophic synergistic denitrification. Figure 4a shows that as the C/N ratio increases, the removal rate of NO_3_^−^-N also increases. Previous studies have shown that when the carbon source is sufficient, there are a large number of denitrifying bacteria, and the denitrification rate is relatively fast. When the carbon source is insufficient, autotrophic denitrifying bacteria are dominant [37]. The growth of these autotrophic denitrifying bacteria is slow, which affects the denitrification process, resulting in an unsatisfactory denitrification process. Traditional sewage treatment technologies require an influent C/N ratio of more than 6–7. In this experiment, iron ore materials were used as fillers, and a good NO_3_^−^-N removal effect was achieved when the C/N ratio was 2. This is because the denitrifying bacteria in the system first use organic matter for heterotrophic denitrification. When the organic carbon source is insufficient, the iron ions and hydrogen ions generated from iron elements are used as electron donors for iron or hydrogen autotrophic denitrification [38]. In addition, iron ions can promote the growth of microorganisms and improve the efficiency of denitrification, thus effectively removing NO_3_^−^-N [39].

#### 3.3.2. PO_4_^3−^-P Removal

As shown in Figure 4b, under different C/N ratio conditions, the effluent PO_4_^3−^-P concentration was lower than 0.25 mg/L, and the average removal rates were 84.41%, 86.07%, and 90.5%. The removal rate of PO_4_^3−^-P by the reactor increases with the increase in the C/N ratio, indicating that the addition of the carbon source can accelerate the degradation of PO_4_^3−^-P. The main reason is that with the increase of the carbon source, the activity of iron autotrophic denitrifying bacteria in the system is enhanced. High-content iron compounds can form precipitates such as iron phosphate with phosphate pollutants, realizing the recovery and utilization of iron-containing sludge and simultaneous nitrogen and phosphorus removal.

### 3.4. Comparison of Different Processes

Table 1 illustrates a comparison of the N and P removal in this study with those in the other literature. Compared with other processes, the denitrification process based on pyrite in this study has the advantages of low operational cost and significant N and P removal effect.

### 3.5. Analysis of Microbial Characteristics

#### 3.5.1. Microbial Abundance and Diversity

During the membrane hanging period, biofilm samples were collected from the lower, middle, and upper parts of the reactor and labeled as A1, A2, and A3, respectively. Under the optimal C/N ratio of 4, the sludge in the lower, middle, and upper parts of the reactor were labeled B1, B2, and B3, respectively. Under the condition of optimal EBCT of 8 h, the sludge in the lower, middle, and upper parts of the reactor were labeled C1, C2, and C3, respectively. Under the optimal pH value of 8, sludge samples were taken from the lower, middle, and upper parts of the reactor and labeled as D1, D2, and D3, respectively. The original sludge was labeled E, and changes in microbial abundance and diversity were analyzed, as shown in Table 2.

As shown in Table 2, the OTU index, Chao index, and Ace index indicate that the bacterial abundance in different parts of different systems is ranked as A2 > A1 > A3, B2 > B1 > B3, C3 > C2 > C1, and D3 > D2 > D1, respectively. The results indicate that there were differences in microbial abundance along the reactor under different operating conditions. Under the optimal C/N condition in the membrane hanging stage, the sludge abundance in the middle of the reactor was the highest, and the sludge abundance in the upper part of the reactor was the lowest, indicating a high number of dominant bacteria in the middle of the reactor. This was mainly because, during the membrane hanging stage and the C/N stage, the reactor was fed from the bottom to the top, with higher nutrient concentrations in the lower part, leading to richer organic matter and more vigorous growth and metabolism of microorganisms. Therefore, the abundance in the middle and lower parts of the reactor is higher. Under the optimal pH and EBCT conditions, the microbial abundance in the upper part of the reactor was the highest, the microbial abundance in the lower part of the reactor was the lowest, and the microorganisms were mainly concentrated in the upper part of the reactor.

The Shannon index showed that the Shannon index of all 13 groups was greater than 4.6, indicating that all samples had rich diversity and bacterial species. The bacterial diversity of the samples was ranked as A2 > A1 > A3; B2 > B1 > B3; C1 > C3 > C2; D1 > D2 > D3. The results show that the order of abundance and diversity of C/N bacteria remained unchanged in the membrane hanging stage, with the highest microbial diversity in the middle of the reactor. This is because the water inlet mode in the upper part of the reactor meant that the nutrients used by microorganisms in the upper part had already been metabolized by microorganisms in the lower part, resulting in the least diversity in the upper part of the reactor. Under optimal pH and EBCT conditions, the microbial diversity along the reactor was different. Under pH conditions, the microbial diversity in the upper part of the reactor was the highest, while the microbial diversity in the middle part of the reactor was the lowest. Under EBCT conditions, the diversity index was opposite to the abundance index, with the highest microbial diversity at the bottom of the reactor and the lowest at the top, indicating that some groups rich in bottom microorganisms dominated.

**Figure 5:**
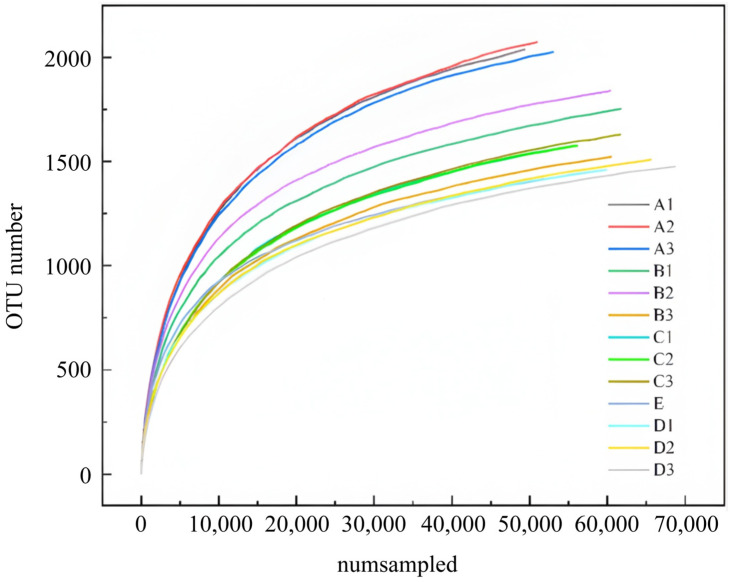
Sparse curve of bacterial Alpha index (taking OTU index as an example).

The serial number of the sample was obtained through microbial measurement. The number of samples extracted was taken as the horizontal axis and the OTU diversity index as the vertical axis. The sparse curve of the bacterial Alpha index under different operating conditions was drawn, as shown in Figure 5. After 70,000 readings of the sample, new bacteria were still detected, representing the authenticity of the bacteria in the sample and the type information of most microbial flora. As can be seen from Figure 5, the number of OTU in group A was the highest, followed by group B, and the number of OTU in group C and group D was less. This may be because, during the membrane hanging period, the high COD concentration in the microbial water provided sufficient nutrients for growth and metabolism, leading to a fast growth and enrichment rate and increased diversity of flora.

Figure 5 shows that microbial community diversity is more complex during the membrane hanging period, demonstrating good biological adaptability, playing a crucial role in the growth, enrichment, and reproduction of the microbial community, and facilitating the long-term and efficient reaction of biological nitrogen and phosphorus removal, resulting in a good removal effect of nitrogen and phosphorus and organic matter in the reactor. In the process of optimizing influencing factors, the bacteria in the reactor gradually utilized Fe^2+^ and S^1−^ in siderite and pyrite as electron donors and NO_3_^−^-N as electron acceptors, converting NO_3_^−^-N into N_2_ under the action of microorganisms. After the completion of membrane attachment, the dominant denitrifying bacteria gradually showed their advantages and continued to grow and multiply, reducing the diversity of the bacteria and increasing the abundance. Due to different microbial growth adaptation conditions, the dominant bacteria of microorganisms vary under the best conditions of C/N ratio, EBCT, and pH value, resulting in different diversities.

#### 3.5.2. Microbial Community Composition

**Figure 6:**
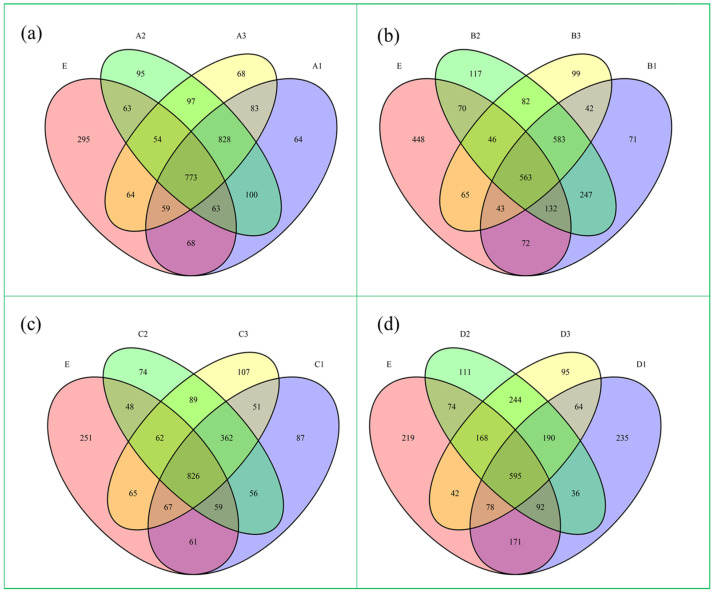
Wayne diagram of species distribution in each ore layer of the reactor when (**a**) the hanging film stage, (**b**) the optimal C/N ratio was 8, (**c**) the optimal EBCT was 8 h and (**d**) the optimal pH condition of 8.

OTU analyses were conducted for the various ore layers at the membrane hanging stage of the reactor and under optimal C/N, EBCT, and pH conditions, respectively. These results were contrasted with the initial sludge sample OTUs. A 97% similarity level was used to select the number of OTU samples for creating a Venn diagram, as shown in Figure 6.

Figure 6a illustrates the high degree of similarity and overlap of the microbial communities between different layers. During the hanging film stage of the reactor, 828 OTUs were shared between layers, while only 64, 95, and 68 OTUs were unique to A1, A2, and A3, respectively. Furthermore, 773 OTUs with the same high species similarity were found in the cross area between each layer and the original sludge in the membrane hanging stage. This indicates that the dominant flora in the original sludge remained active during the membrane hanging stage, proving that the reactor was successfully initiated by the membrane hanging through the trial operation.

Figure 6b depicts the distribution of OTUs in each ore layer of the reactor when the optimal C/N ratio was 8. The number of OTUs shared by multiple layers of the reactor was 583, while the number of OTUs unique to B1, B2, and B3 was 71, 117, and 99, respectively, indicating that the microbial communities between layers were rather heterogeneous at this point. A well-defined C/N setup could allow the reactor microbial population to perform a specific heterotrophic denitrification process while remaining autotrophic, utilizing an external carbon source to carry out a certain heterotrophic denitrification process, thereby meeting the goal of microbial autotrophic/heterotrophic synergistic denitrification. Furthermore, there were 563 OTUs in the cross-zone of each reactor layer with the initial sludge. The similarity began to diminish when compared to the hanging phase. These figures indicate that the reactor produced different dominating flora under different work optimization settings.

Figure 6c illustrates the distribution of OTUs in each ore layer of the reactor when the optimal EBCT was 8 h. The amount of water treated in the system and the impact of contaminants’ degradation were directly tied to the EBCT setting. The figure highlights that 826 OTUs were shared across the reactor’s various levels, whereas 87, 74, and 107 OTUs were specific to C1, C2, and C3, respectively. At this point, there was significant variation in the microbial populations throughout the several levels. This suggests that the microbes in the reactor began to exhibit increased activity under appropriate EBCT, despite a propensity to assimilate to the original sludge. The removal effect was outstanding overall, with phosphate being removed by over 60% and its related NO_3_^−^-N removal rate reaching 90.24%. The significant variation among the various ore layers suggested that the microbial community was vertically distributed under the optimal EBCT. As a result, the distinct reaction areas wrapped up the corresponding functional division, which facilitated the reactor’s development of healthy NO_3_^−^-N and phosphate metabolic pathways.

Figure 6d represents the significant heterogeneity among D1, D2, and D3 under the optimal pH condition of 8, as well as clear variations in the structure of the microbial communities. The figure shows that just 190 OTUs were shared across the reactor’s various levels, whereas the number of OTUs specific to C1, C2, and C3 were 235, 111, and 95, respectively, with a more pronounced stratified distribution. There were 595 OTUs in the superposition area of each layer with the original sludge, indicating a high degree of similarity. In this instance, the equivalent N removal from nitrate (91.23%) was satisfactory. Under the optimal pH conditions, the reactor exhibited a vertical distribution of microbial communities, which was also similar to the initial sludge microbial community.

In summary, optimal NO_3_^−^-N removal generally suggests an acceptable spatial distribution of the reactor’s microbial community, independent of operating conditions. The autotrophic denitrification of microorganisms requires the proper pH and EBCT, and with the optimal C/N setting, works in tandem with the heterotrophic denitrification process to handle NO_3_^−^-N. All of the procedures above are carried out in the metabolic process of microorganisms. Therefore, the secret to increasing the reactor’s treatment capacity is to create an environment conducive to microbial survival.

The overall makeup of the microbial community structure and the variation between the reactor’s various substrate layers throughout the hanging phase and under optimal C/N, EBCT, and pH settings, respectively, were comprehended using the OTU analysis. The experiment was conducted to analyze several samples in depth at the microbiological portal level in order to gain a better understanding of the distribution and organization of the microbial community under various operational conditions.

*Proteobacteria* were the primary phylum in the reactor. The percentage of *Proteobacteria* was maintained in the range of 20–40% in each mineral layer of the reactor during the hanging phase and under various optimized settings of C/N, EBCT, and pH, indicating that the reactor performs a steady denitrification process. In this study, *Bacteroidota* were more common and in varied stages of development during this experiment. *Bacteroidota* had the same abundance ratio in all layers of the reactor during the membrane stage, but they were primarily enriched in the middle and top layers of the system at optimal C/N, EBCT, and pH conditions. Additionally, the quantity of *Bacteroidota* dropped in all other settings compared to the original sludge, indicating that *Bacteroidota* were not the dominating phylum during reactor operation. The larger abundance corresponds to the retention of the original sludge in the reactor. *Chloroflexota* was abundant under optimal EBCT and pH settings, accounting for 20–60% of the microbial content in the reactor, and it is a microflora that should not be overlooked during the reactor’s nitrogen removal process. This could also explain why the above two circumstances result in better NO_3_^−^-N elimination. It was discovered that *Chloroflexota* was closely linked to SO_4_^2−^ generation in sulfur autotrophic denitrification [45]. Since the primary objective of optimization under optimal C/N conditions was the availability of external carbon sources, the *Chloroflexota* content was not noticeable in abundance in B1, B2, and B3.

**Figure 7:**
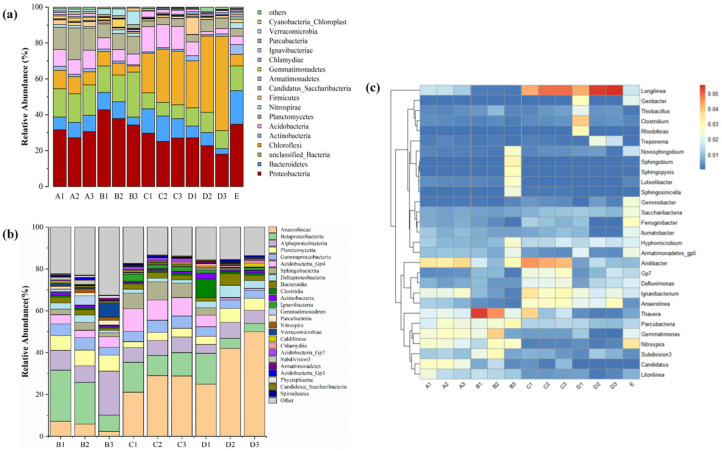
Analysis of microorganisms in the samples (**a**) abundance at the community phylum level, (**b**) relative abundance at the class level, and (**c**) heat map of dominant genus at the genus level.

Figure 7a shows the relative abundance of microbial community structure in each mineral layer of the reactor during the hanging phase and under various optimized C/N, EBCT, and pH parameters. *Proteobacteria*, *Bacteroidota*, *Gemmatimonadota*, *Acidobacteriota*, *Actinomycetota*, and *Bacillota* were the dominant microflora in the device, accounting for around 70–90% of the microbial content of each sample. *Proteobacteria* are common in wastewater treatment procedures, and their abundance corresponds to the more efficient purifying capacity of wastewater treatment facilities [46]. Furthermore, the reactor’s microbial population also includes *Verrucomicrobiota*, *Chloroflexota*, *Nitrospirota*, *Bacillota*, and *Chlorobiota*, all of which have a low abundance.

Furthermore, at an EBCT of 8 h, *Chlorobiota* were present in around 20% of the population and frequently only emerged during the sulfur autotrophic reaction. A few of these species were linked to the sulfur autotrophic denitrification process and could play a significant role in the reactor’s NO_3_^−^-N breakdown. In the reactor, several other phyla with lower abundance should not be disregarded. The taxa known as *Acidobacteriota* are typical of manmade wetlands and filter ponds [47]. *Nitrospirota*, the primary nitrite-oxidizing bacteria, is linked to the N, CH_4_, and S cycles and aids in the development of robust biochemical cycling processes in the reactor [48]. Numerous heterotrophic nitrifying bacterial taxa are found in *Actinomycetota*, which tend to grow dominant in locations with a lot of organic materials [49]. The microbial community of the reactor in this study is made up of various phyla, which work together to effectively remove contaminants from the reactor.

During the hanging period and under various process settings, the reactors were examined at the microbial genus level on the filler. Figure 6b,c display the relative abundance of the dominating bacteria at the class and genus levels.

At the class level, the most common bacterial orders in the reactor were *Betaproteobacteria* (up to 29%), *Acidobacteria*_Gp4 (up to 10.8%), *Bacteroidia* (up to 5.4%), and *Nitrospiria* (up to 3.14%), as shown in Figure 7b. The genus-level study shows that the reactor had more denitrifying bacteria. Nonetheless, the majority of denitrifying bacteria were members of the *Betaproteobacteria* [50], which has the highest *Proteobacteria* phylum abundance. Additionally, it is evident that, while the microbial abundance varied at the class level, the microbial community structure was identical at various depths. This is mostly because, although the reactor received the same influent water, the abundance changed depending on the depth because of variations in the nutritional composition and ambient pressure [51]. The NO_3_^−^-N removal was 98.78% at a C/N of 4, indicating that the system’s denitrification ability was improved by the rise in *Betaproteobacteria* order abundance.

At the genus level, *Longilinea*, *Thauera*, *Aridibacter*, *Rhodoplanes*, *Thiobacillus*, and *Nitrospira* were the most dominant genera in the various samples, as shown in Figure 7c. *Longilinea* had the highest abundance share at 20.4%, followed by *Thauera* at 17% and *Aridibacter* at 7.6%. *Longilinea*, a purely anaerobic microbe, can break down a variety of carbohydrates [52] and eliminate organic contaminants from wastewater. In the system dosed with sodium acetate, the abundance of *Longilinea* was greater in D2 and D3 than in the original activated sludge samples and D1. This is primarily due to the top-down influent strategy, which allows the microbes in the middle and bottom areas to absorb more organic matter while *Longilinea*, which is most abundant, can develop in an environment with low oxygen levels. Denitrifying bacteria such as *Thauera*, *Aridibacter*, and *Gemmatimonas* are crucial to the denitrification process [53]. The reactor had a high NO_3_^−^-N and NO_2_^−^-N removal capability in conjunction with these denitrifying microorganisms. The figure shows that, when the EBCT was changed, the lowest C1 sample of the reactor had the largest abundance of *Thauera*. This indicates that denitrification primarily takes place in the middle and lower sections of the reactor, which is the primary location for the removal of NO_3_^−^-N.

*Thiobacillus* is a significant group of bacteria in the reactor that are involved in the sulfur autotrophic denitrification and denitrogenation process. *Thiobacillus* species can oxidize sulfur to SO_4_^2−^, and their abundance is highest at the lower level of the reactor where the NO_3_^−^-N content is highest and the bindable electron donor (nitrate-nitrogen) of *T. denitrificans* is increased [54]. The analysis revealed that the highest abundance of *Thiobacillus* was 1.1% at pH 7–8. Conversely, *Rhodoplanes* is a genus that can use sulfurate and sulfuride as electron donors to support its growth and metabolism. When the reactor was operating, the amount of *Rhodoplanes* was much higher than in the samples from the initial activated sludge and the hanging membrane. Specifically, during the EBCT stage of 12 h at pH 7–8, the largest abundance of *Rhodoplanes* was detected in C1, C2, and C3, with 1.2%, 1.3%, and 1.7%, respectively. This suggests that throughout this process, *Rhodoplanes* absorbed and used the SO_4_^2−^ generated in the reactor for growth and reproduction. The reactor was found to have been colonized by *Geobacter*, a type of anaerobic bacterium that can reduce Fe^3+^ by utilizing NO_3_^−^-N as an electron acceptor [55]. The figure illustrates that the anoxic lower (D1) and middle (D2) layers had larger abundances of *Geobacter* spp., 1.4% and 1.9%, respectively. At the same time, the reactor’s P removal rate increased to 70–90%, suggesting that the reactor’s Fe^2+^ and Fe^3+^ ions interacted with the phosphate to generate FePO_4_ precipitate.

In conclusion, the system had high denitrification efficiency under the combined action of denitrifying bacteria. The reactor had a large number of anaerobic bacteria, particularly in the bottom and middle zones, and the anoxic environment and higher nutrients encouraged the growth of denitrifying bacteria. The identification of *Thiobacillus*, *Rhodoplanes*, and *Geobacter* suggested that bacteria absorbed the sulfur-containing compounds and iron ions in iron ore for their development and metabolism.

### 3.6. Prediction of Microbial Community Functions

FAPROTAX is a functional annotation database that analyzes the functions of microbial communities based on prokaryotic microorganism classification. It contains functional annotation information such as nitrate respiration, methanogenesis, and fermentation. The functions predicted by FAPROTAX mainly focus on the cycling of sulfur, carbon, hydrogen, and nitrogen. In this section, the data on the prediction of microbial community functions during the biofilm formation stage of the reactor, as well as under the optimal conditions of C/N, EBCT, and pH, are summarized (Table 3).

As shown in Table 3, the microbial community in the reactor exhibits a rich variety of functional categories related to organic matter degradation and nitrification–denitrification for nitrogen removal. Among them, the methanotrophy function is associated with pollutant degradation and usually occurs in places with a high nitrite level [56]. The methanotrophy values generally fluctuate between 100 and 800, indicating that each ore layer has a good nitrite metabolism ability. Methylotrophy refers to the ability to utilize reducing one-carbon compounds, such as methanol, as the sole carbon source and energy source [57]. This function showed good numerical performance under various working conditions.

As an important S-autotrophic function in the reactor, in terms of functional prediction, it was manifested as sulfate respiration [58], sulfur respiration [59], and respiration of sulfur compounds [60]. Most of the microorganisms with the sulfate respiration function can couple the oxidation of organic acids or ethanol with the reduction of sulfate, sulfite, or thiosulfate. NO_3_^−^-N can be regarded as an alternative electron acceptor for chemolithoautotrophy of microorganisms with sulfur respiration function under anoxic conditions, thus removing NO_3_^−^-N in sewage [59]. Most of the above functions had relatively high values in various stages of the reactor. However, the sulfur respiration function had relatively low values in each stage, indicating that the reactor has poor sulfur respiration ability but good utilization ability for sulfate and other sulfur compounds. In addition, the microbial community functions related to nitrogen removal also included nitrogen fixation, nitrate reduction, anammox, nitrate denitrification, nitrite denitrification, and nitrous oxide denitrification. These functions had relatively high values (100–400) during the C/N optimization stage in the reactor, indicating that the reactor has a stable nitrogen removal process.

## 4. Conclusions

In this study, the N and P removal efficiencies and denitrification pathways in an autotrophic denitrification biofilter packed with natural pyrite under various operating conditions were comprehensively investigated. The results demonstrated that the performance of N and P removal was significantly influenced by the EBCT. Specifically, an EBCT of 8 h was identified as optimal for achieving the highest denitrification and PO_4_^3−^-P removal efficiencies, with a stable PO_4_^3−^-P removal rate exceeding 60%. The lowest PO_4_^3−^-P concentration in the effluent was observed at an EBCT of 12 h.

The study also elucidated the mechanisms of N and P removal at different pH levels. At pH 5, the formation of FePO_4_ facilitated a maximum PO_4_^3−^-P removal rate of over 95%. In contrast, at pH 9, the majority of PO_4_^3−^-P removal was attributed to the precipitation of Fe(OH)_3_. Additionally, the removal rate of PO_4_^3−^-P exceeded 90% at pH values between 7 and 8, indicating that this pH range is optimal for nitrate reduction.

Furthermore, the addition of an external C source was found to enhance synergistic autotrophic and heterotrophic denitrification. The optimal N and P removal efficiencies were achieved at a C/N ratio of 4. As the C/N ratio increased, the proportion of heterotrophic denitrification also increased, highlighting the importance of balancing the C/N ratio for efficient nutrient removal.

Microbial community analysis revealed that the microbial populations in different sections of the reactor were highly similar and overlapping. At the phylum level, the dominant microbial groups included *Proteobacteria*, *Bacteroidota*, *Gemmatimonadota*, *Acidobacteriota*, *Actinomycetota*, and *Bacillota*. *Chlorobiota*, *Bacteroidota*, and *Chloroflexota* were identified as key contributors to both heterotrophic and autotrophic denitrification processes. At the genus level, *Longilinea*, *Thauera*, *Aridibacter*, *Rhodoplanes*, *Thiobacillus*, and *Nitrospira* were the most dominant genera. *Thauera*, *Aridibacte*, and *Gemmatimonas* played crucial roles in heterotrophic denitrification, while *Thiobacillus*, *Rhodoplanes*, and *Geobacter* were associated with autotrophic denitrification.

Overall, this study provides valuable insights into the operational parameters and microbial mechanisms underlying efficient N and P removal in autotrophic denitrification biofilters packed with natural pyrite. The findings highlight the importance of optimizing EBCT, pH, and C/N ratios to enhance nutrient removal performance and elucidate the key microbial taxa involved in the denitrification process. The denitrification process based on pyrite can effectively achieve denitrification and nitrogen removal in wastewater with a low C/N ratio, significantly reducing the TN concentration in the effluent. Compared with the traditional heterotrophic denitrification process, this process has the advantages of low treatment cost and significant effect. This study provides a theoretical basis for the efficient nitrogen removal treatment of wastewater and has important practical application value.

## Figures and Tables

**Table 1:** Comparison of different processes.

Process	Results	Comparisons	Reference
Pyrite-based denitrification combined with electrochemical disinfection	At a hydraulic retention time of 18 h, a nitrate removal efficiency of 79% was achieved with an initial nitrate concentration of 178 mg/L NO_3_^−^.	This study achieved a higher denitrification efficiency while exhibiting significantly lower energy consumption compared to study of Ntagia et al.	[40]
Biochar-pyrite vertical flow constructed wetlands	At a biochar-to-pyrite volume ratio of 1:1, Highest TN removal reached at 86.0 ± 2.5%. No optimal biochar-to-pyrite ratio exists to peak TP removal.	In this study the P removal was higher and needed lower carbon source.	[41]
Aerated biofilter driven by sponge iron	The results showed ammonia removal efficiency reached 94.1% and total inorganic nitrogen removal efficiency was up to 70.6% at HRT of 19 h and gas–water ratio of 18.	The N removal was lower than this study, and it might need more energy for aeration.	[42]
Moving bed biofilm reactor	The optimum choice of C/N ratio for nitrogen removal by denitrification MBBR was 4.6.	The optimal C/N ratio of this study was 4, which was lower while the removal of N was higher.	[43]
Pyrite/PHBV mixotrophic denitrification system	The removal efficiency of nitrogen and phosphorus was 96% and 25%, with low sulfate production.	Compared with study of Zhou et al., this study showed high phosphorus removal, strong resistance to shock loads, and low operational costs.	[44]

**Table 2:** Alpha diversity index under different systems.

Sample	OTUs	Chao	Ace	Simpson	Shannon	Coverage
A1	2038	2418.3208	2383.0271	0.0075	5.9269	0.9908
A2	2073	2477.6946	2437.3617	0.0086	5.9390	0.9909
A3	2026	2383.2749	2330.4918	0.0084	5.8709	0.9919
B1	1753	2044.6811	2003.0342	0.0307	5.3508	0.9943
B2	1840	2167.6146	2110.4507	0.0113	5.7408	0.9939
B3	1523	1818.5789	1812.5949	0.0135	5.3331	0.9942
C1	1569	1896.4264	1843.5595	0.0204	5.0978	0.9933
C2	1576	1852.0476	1827.5043	0.0294	4.9609	0.9939
C3	1629	1933.4663	1919.5652	0.0257	5.0537	0.9940
D1	1439	1765.9856	1681.0433	0.0117	5.5351	0.9946
D2	1461	1826.9272	1776.9442	0.0220	5.1596	0.9941
D3	1510	1806.4188	1789.8089	0.0427	4.9013	0.9948
E	1476	1845.8095	1781.8664	0.0540	4.6825	0.9948

**Table 3:** Prediction of microbial community functions.

Group	A1	A2	A3	B1	B2	B3	C1	C2	C3	D1	D2	D3	E
methanotrophy	754	632	598	568	513	470	87	134	138	110	101	313	222
methylotrophy	754	633	598	570	517	956	95	136	139	110	105	317	338
aerobic_ammonia_oxidation	11	14	10	5	83	30	41	41	35	86	188	42	142
aerobic_nitrite_oxidation	772	689	467	369	1191	996	36	45	42	69	24	52	1764
nitrification	783	703	477	374	1274	1026	77	86	77	155	212	94	1906
sulfate_respiration	246	253	247	277	343	71	82	100	85	375	1201	892	131
sulfur_respiration	2	9	8	27	9	8	7	7	8	282	13	9	10
respiration_of_sulfur_compounds	249	262	256	304	354	80	91	107	94	658	1216	901	142
anammox	855	908	649	139	378	57	77	20	197	2	4	3	0
nitrate_denitrification	0	1	2	95	311	0	4	2	2	1	0	0	2
nitrite_denitrification	0	1	2	95	311	0	4	2	2	1	0	0	2
nitrous_oxide_denitrification	0	1	2	95	311	0	4	2	2	1	0	0	2
denitrification	0	1	2	95	311	0	4	2	2	1	0	0	2
nitrogen_fixation	39	53	57	302	62	64	170	189	236	87	183	294	225
nitrite_respiration	1325	1468	1097	10,723	5566	706	2347	236	643	71	34	46	123
dark_sulfide_oxidation	42	41	64	94	198	51	40	85	115	624	89	98	10
dark_oxidation_of_sulfur_compounds	44	43	65	107	202	55	83	97	148	642	93	101	19
aerobic_chemoheterotrophy	1952	1973	2208	12,725	6481	10,502	5985	2936	3822	3947	2112	2048	4458
hydrocarbon_degradation	766	638	606	583	518	476	105	138	140	116	102	314	223
nitrate_respiration	564	679	531	10,945	5314	774	2438	363	707	181	174	104	1006
nitrate_reduction	612	742	591	11,047	5661	873	2502	418	762	244	247	193	1089
nitrogen_respiration	1419	1587	1180	11,084	5692	831	2515	383	904	183	178	107	1006
chemoheterotrophy	3106	2966	3172	13,795	7770	11,800	6506	3544	4427	6098	2930	3074	5377
sulfite_respiration	5	2	2	7	19	4	7	6	8	16	39	68	32
thiosulfate_respiration	3	9	9	27	11	9	9	7	9	285	15	9	16

## Data Availability

The data presented in this study are available on request from the corresponding author.
